# Are neuroinflammation and citokines possible novel targets for therapeutic treatments?

**DOI:** 10.1192/j.eurpsy.2025.252

**Published:** 2025-08-26

**Authors:** P. Falkai

**Affiliations:** Psychiatry, Munich University Hospital, Munich, Germany

## Abstract

**Abstract:**

Schizophrenia is a serious mental illness with positive, negative and cognitive dysfunctions and a significant deterioration in psychosocial functioning. Interactions between genetic predisposition and environmental stressors at the early stages of life, and subsequently a molecular level neurodegeneration process are important in the development of schizophrenia. Current approaches suggest that cytokines-induced neuroinflammation might have a role in the development of several psychiatric disorders, including schizophrenia.

**Disclosure of Interest:**

None Declared

